# Social Interoception and Autonomic System Reactivity during Synchronization Behavior

**DOI:** 10.3390/bs14030149

**Published:** 2024-02-20

**Authors:** Michela Balconi, Laura Angioletti

**Affiliations:** 1 International Research Center for Cognitive Applied Neuroscience (IrcCAN), Università Cattolica del Sacro Cuore, 20123 Milan, Italy; 2 Research Unit in Affective and Social Neuroscience, Department of Psychology, Università Cattolica del Sacro Cuore, 20123 Milan, Italy

**Keywords:** social interoception, biofeedback, autonomic measures, synchronization

## Abstract

Background: Within the social interoception field, little is known about the impact of interoception on autonomic system reactivity during synchronization tasks. The impact of social framing manipulation and Interoceptive Attentiveness (IA; defined as concentrated attention on the breath for a specific time interval) on autonomic responses during interpersonal synchronization was investigated in this research. Methods: Under two experimental interoceptive conditions—the concentration and no focus on the breath condition—participants completed two synchronization tasks. A social framing was given to participants by informing them that they needed to complete the tasks in unison to improve their collaboration abilities. Autonomic responses (electrodermal activity and cardiovascular indices) were collected throughout task performance. Results: Two orders of results were observed: high cognitive engagement was detected during the focus on the breath condition and for the social frame. This effect was specifically observed for the motor compared to the linguistic synchronization task. Meanwhile, a potential lack of emotional control was observed in the no focus on the breath condition when the synchronization tasks were not socially framed. Conclusions: Such results encourage the use of the hyperscanning paradigm to deepen the impact of IA in real-time and ecological interpersonal synchronization dynamics.

## 1. Introduction

The perception of visceral stimuli originating inside of the body represents a core component of homeostasis maintenance and bodily self-experience, and it is known as interoception [1], which is a multidimensional perception of internal states of bodily arousal. It represents an important source for an individual’s body representation and has been linked to a variety of psychological functions, such as emotion processing, social cognition, and self-awareness [1].

Interoception is a multicomponential construct including multiple dimensions ranging from the basic bodily signals perception to the cognitive level. For instance, interoceptive sensitivity (IS) is a first dimension referring to the individual’s capability to perceive and accurately refer one’s body signal (such as counting the heartbeat) during resting state, whereas interoceptive awareness (IAw) refers to the individual’s confidence and awareness on the accuracy in perceiving his/her body signals (i.e., characterizes the metacognitive representation of one’s own interoceptive abilities) [2,3]. These dimensions both vary because of individual differences in perceptual abilities. 

Indeed, prior research suggested that these interoceptive dimensions (i.e., IS and IAw) are a generally stable characteristic that influence participants’ ability to discriminate “self” from “other” in the empathic resonance of action as well as their subjective experience of emotion [4]. On the other hand, recent works revealed that these interoceptive dimensions can be modified by specific trainings (such as slow breathing, mindfulness training, vagus nerve stimulation) and, in turn, this might have a positive impact on cognition, emotion and clinical outcomes [5,6]. Between the other interoceptive dimensions, the intentional modulation of attention toward a specific bodily information for a fixed timed interval, that is interoceptive attentiveness (IA) [7], can be trained even through simple breath awareness tasks and might impact on individual [6] and social processes [8,9].

In fact, interoception has been now widely demonstrated to play a role not only in processes that concern the individual and his body as well as in a range of social phenomena such as the differentiation between the self and the other [10], social cognition [11], loneliness and relatedness [12] and empathic responses [13]. In line with these studies, it has been recently demonstrated how IA can impact on the neural correlates implicated in basic interpersonal synchronization tasks [8,9,14]. For instance, when a joint task is performed and the individual focuses on his/her physiological body reactions, the brain hemodynamic correlates are “boosted” in neuroanatomical regions that support sustained attention, reorientation of attention, social responsiveness, and synchronization (i.e., the prefrontal cortex, PFC) [8]. Furthermore, the PFC responds significantly more as the person consciously focuses on physiological interoceptive correlates and performs a motor task requiring synchronization, particularly when the task is socially framed [8]. On the other hand, the right PFC was shown to be more involved when an explicit focus on the breath was induced during the cognitive linguistic task requiring synchronization with a partner: interestingly, this right lateralized effect was not significant for the motor task [9]. In addition, two main patterns of electrophysiological frequency band modulation related to mental concentration, coordinated, and controlled motor activity were found during the execution of a motor compared to cognitive synchronization task while a person is focusing the attention on one’s breath [14]. 

Following the evidence obtained from these studies, the goal of this research is to determine whether the Autonomic Nervous System (ANS) response may be affected by the intentional attention on one’s interoceptive correlates (IA) during simple social synchronization activities.

Indeed, there is no extensive evidence within the “social interoception” research field on how interoceptive and social context manipulation affect the ANS correlates [12].

In a previous study, Ferri and colleagues (2013) exploited a realistic social interaction task and showed how, through the activation of various ANS mechanisms, IAw concurs to individual variability in interpersonal predisposition and the spatial mapping of social relations [15]. During the task, Respiratory Sinus Arrhythmia (RSA) was evaluated by the authors in a social as well as a non-social condition. For the social condition, each participant observed the experimenter’s hand performing a caress-like motion from various distances away from participant’s own hand. The experimenter’s hand was replaced by the movement of a metal stick for the non-social condition. The RSA is one of the heart rate variability (HRV) periodic constituents [16] directly resulting from the interaction between the cardiovascular and respiratory systems [17]. RSA is an index of social disposition [18] and positive social functioning both in healthy [19] and clinical samples [20,21], and it can be modulated by emotional processing [22]. According to the findings, only participants with higher IAw levels demonstrated more robust autonomic reactions in a social condition as opposed to a non-social condition. Conversely, participants with low IA levels were less likely to engage in social interactions because it took more intrusive social stimuli—like touching their hand— to successfully predispose their autonomic reaction to these stimuli [15]. Also, in another work, higher interoceptive accuracy was previously related to higher social disposition (higher RSA at rest and during social proxemics stimuli exposure) in healthy controls compared to clinical samples [23]. 

Although these studies have considered the RSA compound index, other cardiovascular indices can be taken into consideration. For instance, a recent study explored the relation between IA manipulation and ANS response in the context of empathy for pain [24]. IA was manipulated by requiring healthy participants to focus on their breath during stimuli presentation, while the control group did not receive any direct interoception instructions. Participants were asked to watch stimuli that belonged to different body parts—such as the hand vs. the face—that were either unpleasant or not and that were exhibited in social or individual settings (i.e., the single body part receiving the painful stimulation versus the interaction between two people in which one of them inflicts painful stimulation on the body part of the other). For both groups, higher Pulse Volume Amplitude (PVA) values marked a physiological reaction of engagement toward the perceived effect of physical pain. Interestingly, for the interoceptive group, an increase in inter-beat interval (IBI) marked of greater autonomic self-regulation for painful facial stimuli [24].

Although they can provide interesting information on the ability to cope with social situations, no previous studies to date explored a wide range of ANS indices in the context of interoception and social synchronization. 

As far as social context manipulation is concerned, a former study [24] demonstrated that interoceptive manipulation impacts on ANS correlates, while a scenario with a high emotional and empathic impact is shown; however, no significant differences were found for the manipulation of the social context. On the other hand, by using different manipulations of the social context, several studies observed significant differences in ANS metrics. For instance, Singleton and colleagues [25] used human versus non-human emotional stimuli (corresponding to social and non-social stimuli), and they discovered that in healthy individuals scoring highly on the autistic symptoms scale, Skin Conductance Responses (SCRs) increase for socially relevant cues than for those without social relevance. Also, previous works on cooperation during social interactions found that Skin Conductance Level (SCL) and SCR indices rose along with HR in high emotional engagement scenarios where feedback reinforcing the favorable results of the cooperative effort of the intersubjective exchange was used to create a cooperative goal [26]. According to research on Electrodermal Activity (EDA), both SCL and SCR are accurate physiological measurements of emotional and empathic responsiveness [27], and measuring it during social interaction is a particularly sensitive predictor of arousal [28,29]. However, no previous studies explored the effect of interoception on the ANS during an interactive social situation that requires interpersonal synchronization and in which the shared intentionality is clearly emphasized. 

Given these premises, we were interested in examining the role of IA manipulation in fostering physiological synergy between individuals, particularly by increasing its efficacy in interactional contexts [12]. Motor and language synchronization tasks have been a common experimental paradigm for simulating interaction exchanges in the past [30] In this investigation, we choose to employ two basic motor and speech synchronization tasks that comprise modified versions of the finger-tapping task [31] and the alternate speech task [32], respectively. The synchronization task that the participants completed (motor versus linguistic) as well as the presence and absence of interoceptive focus—that is, when the participants’ attention was focused on the breath versus not—were both examined in this experimental design. Moreover, the social context manipulation was operationalized by stressing the shared intentionality during the synchronization tasks (i.e., by applying or not a social frame to the synchronization task).

Thanks to this experimental approach, this research aims at investigating the impact of social framing manipulation and IA on autonomic responses during interpersonal synchronization. 

First, considering prior research on the manipulation of IA, it is hypothesized that the execution of synchronization tasks during the focus compared to the no focus on the breath condition will lead to higher cardiovascular indices of variability (such as IBI and HRV) that are associated with better autonomic self-regulation and the effectiveness of coping mechanisms [33].

Secondly, regarding the manipulation of the social context, it is expected to find higher EDA (SCL and SCL) and HR values, as predictor of arousals and positive engagement due to emotional and empathic responsiveness [26,28], in the socially framed condition—that is, when the shared intentionality is stressed compared to the non-socially framed condition.

Thirdly, it may be expected to observe such significant differences in autonomic reactivity especially when participants will be performing the motor as opposed to the linguistic synchronization task given the effect of the social frame we previously observed specifically for the motor synchronization task in our previous research [14].

## 2. Materials and Methods

### 2.1. Sample

The present research included 33 Caucasian university students recruited through a non-probabilistic convenience sample method (19 males and 14 females; total sample Mean (M) age = 27.1; Standard Deviation (SD) age = 3.19). Since the phenomena under study is relatively new in the field of social neuroscience and the literature did not provide systematic repeated evidence, it was not feasible to use previous references to predict the extent of the expected significant effects. Thus, to estimate a minimum required sample size, we ran a priori power analysis for repeated measures ANOVA, and a total sample size (with alpha error probability = 0.05 and power 0.80) of 28 was the minimum for detection of a significant within effect or interaction between factors (G*Power 3.1 software, Heinrich-Heine, Germany [34]).

Past meditation expertise, severe physical and chronic illnesses, traumatic brain injury, seizures, and psychological, psychiatric, and neurological disorders were considered as exclusion criteria. We followed these criteria as in previous studies on interoception focused on healthy samples [8,9]. No neurocognitive deficits, neurological or mental disease history, or current pharmaceutical medication that may change neurofunctional responses or affect cognition or judgment were present in any of the included participants. Also, all subjects included in the study were right-handed, had normal or corrected-to-normal eyesight, normal global cognitive functioning and no habitual drug taking. Written informed consent forms were signed by participants who willingly agreed to participate in the study; no economic reward was given for their contributions. This study was approved by the Ethics Committee of the Department of Psychology of the Catholic University of the Sacred Heart in Milan, Italy (approval code: 2020 TD-a.a.2020–2021). The research was carried out following the principles of the Declaration of Helsinki (2013).

### 2.2. Joint Synchronization Tasks

Two joint tasks were adopted in the current experiment: simple motor and linguistic synchronization tasks. These tasks have been previously utilized and fully described in other functional Near-Infrared Spectroscopy (fNIRS) and electroencephalographic (EEG) research [8,9]. They were chosen to maintain the consistency in the experimental design for this study on autonomic indices.

The motor synchronization task consisted of the participant synchronizing and coordinating their finger-tapping movements for three minutes with the experimenter. In particular, the participants in the motor task were told to put their hands on the table in the prone position with their elbows resting on the surface and their fingers spaced about one centimeter apart. They were told to raise the fingers of their dominant hand and tap the table with their thumb, index, middle, ring, and little fingers. They were just told to coordinate with the movement made by the researcher who was seated in front of them; they were not told to lift their fingers as high as possible or to perform this movement at a specified tempo. The full description of this motor synchronization task can be found in a previous study [8]. For this study, a finger-tapping pattern was counted from start to finish with an average of 60 loops being recorded.

The linguistic synchronization task consisted of a modified version of the human-to-human alternating speech task, requiring the participant to syllabicate in unison for three minutes with the experimenter. The participant in this modified version of the alternating speech task was instructed to pronounce the four syllables “LA”, “BA”, “CA” and “DA” in a sequential and alternating manner. For example, the experimenter would say “LA” and the participant would respond with “LA” and so on. The speech’s rhythms were not decided upon beforehand. Without breaks, each language synchronization task session lasted three minutes. The full description of this linguistic synchronization task can be found in a previous study [9]. In this study, a linguistic repetition pattern was counted from start to finish with an average of 45 loops being recorded.

### 2.3. Procedure

Participants received procedural instructions prior to the experiment stating the execution of the two joint synchronization tasks using IA manipulation under various experimental conditions. The first condition consisted of the participants focusing on their breath in order to control IA. Participants were asked to focus on their breathing during this assignment. The following instructions were given: “While you perform the task, try to pay attention to how you’re feeling and whether your breathing changes”. Participants were not told to breath at a particular pace. In the control condition (with no interoception manipulation) no specific instructions were given, and individuals were only required to execute the joint tasks. 

For the social framing, participants were asked to better synchronize so that they could improve teamwork skills while performing the same motor and linguistic synchronization tasks. In this way, the social frame was introduced by emphasizing the shared intentionality [8]. To avoid any potential biases caused by sequence effects, the order of the condition and the synchronization tasks was randomized and counterbalanced.

The synchronization tasks and the condition were counterbalanced and randomly ordered.

**Figure 1:**
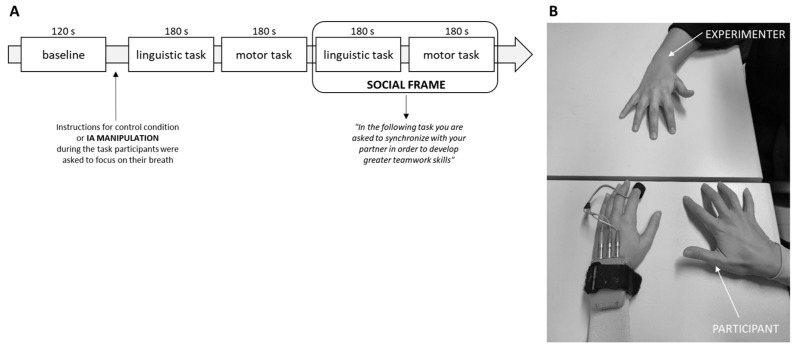
Description of the experiment procedure. (**A**) Experimental procedure representing the conditions and timing for the execution of the synchronization tasks and (**B**) the autonomic measures recording from the participants while performing the motor synchronization task. The task execution was randomized and counterbalanced for the task type and condition in order to prevent the order effect.

During the debriefing phase that followed the experiment, the manipulation of IA was checked: participants rated their level of breath attention on a Visual Analogue Scale from 0 to 10 and their self-perceived sense of synchronization. “From 0 to 10, how much attention did you pay to yourself throughout the activity?” was the question that participants were asked to rate the amount of attention they gave to themselves during the task. For the focus condition, the average score for every participant exceeded five points (M = 8.75; SD = 1.08), whereas mean values were lower in the no focus condition (M = 5.97; SD = 1.54). To preserve the procedure’s consistency, the same interoceptive manipulation was applied in earlier works [9]. Also, at the end of each task, individuals expressed feeling synchronized, which was at 80% for each experimental condition. The experiment lasted approximately an hour (Figure 1A,B).

### 2.4. Autonomic Data Recording

An X-pert2000 wearable Biofeedback systems with a MULTI radio module (Schuhfried GmbH, Modling, Austria) was used for the autonomic activity gathering and recording. A sensor was attached to the second finger of the non-dominant hand’s distal phalanx in order to collect the signal. It enables the measurement of SCL, SCR in lS, and HR in beats per minute (bpm). A current–current measurement was used to record the SCL value with an EDA gold electrode at a sampling frequency of 2 kiloHertz (kHz). Polarization was reduced using alternating voltage. With a sampling frequency of 20 Hz, the SCL calculation has a measure resolution of 12 nanoseconds (ns). Photoplethysmography was used to assess PVA, BVP, and HR at a sampling frequency of 500 Hertz (Hz). In order to prevent hand movements from interfering with the recordings, a sending unit’s accelerometer in meter/square second (m/s^2^) was used to track the movement of the non-dominant hand. 

Following the inspection of qualitative and quantitative data to identify and minimize recording (motor) or biological artefacts, both standard measures of cardiac activity (HR, inter-beat interval (IBI)) and a measure of HR variability (the standard deviation of IBI) were computed to obtain stress-related cardiac responses as well as a measure of vagal tone, which is connected to the functionality of parasympathetic recovery mechanisms that promote the body’s return to homeostasis. Before the beginning of the tasks, we recorded a 120 s baseline of participant’s autonomic responses at the resting state.

### 2.5. Statistical Data Analysis

Repeated measures ANOVAs with Condition (2: focus, no focus) × Task (2: motor, linguistic) × Frame (2: no social, social) as independent within factors was applied to autonomic data (SCR, SCL, BVP, PVA, HR, IBI and HRV). In case of significant effects in the data, pairwise comparisons were performed. Pairwise comparisons were utilized to confirm the simple effects of significant interactions, and Bonferroni correction was applied to minimize the potential biases associated with repeated comparisons. When necessary, the degrees of freedom for each ANOVA test were adjusted using the Greenhouse–Geisser epsilon. Additionally, kurtosis and asymmetry indices were checked in order to make a preliminary assessment of the data distribution’s normality. All the assumptions required for ANOVA test were met. The size of statistically significant effects has been estimated by computing partial eta squared (*η*^2^) indices.

## 3. Results

### 3.1. First Order of Results

#### 3.1.1. SCL and SCR

**Figure 2:**
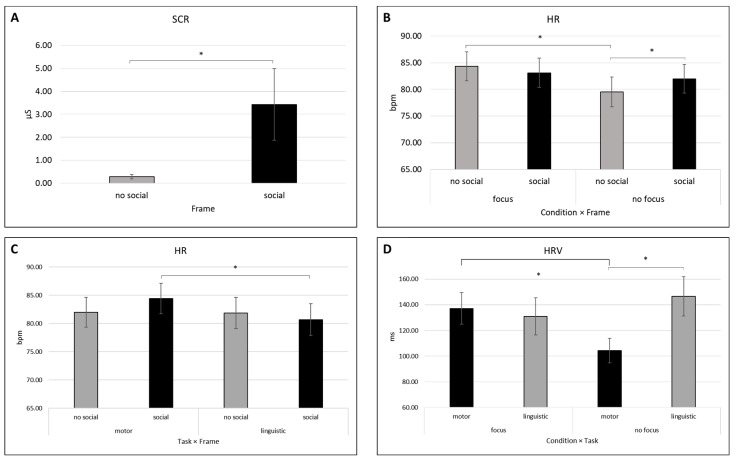
First order of autonomic results: SCR, HR and HRV. (**A**) The bar graph displays higher SCR values for the social compared to no-social frame condition. (**B**) The bar chart displays significantly greater HR values in the focus condition confronted with the no focus condition in the no social frame and significantly higher mean values in the social frame compared to the no social frame in the no focus condition. (**C**) Greater HR values in the motor task compared to the linguistic task when performed in the social frame condition can be observed in the graph. (**D**) The graph shows significantly higher HRV values in the focus condition compared to the no focus condition in the motor task and significantly higher mean values in the linguistic compared to the motor synchronization task in the no focus condition. Bars represent the standard error (SE) of ±1 for all plots; asterisks (*) denote statistically significant differences with *p* < 0.05.

For the SCR index, a significant main effect was identified for Frame (*F* [1, 27] = 4.400, *p* = 0.045, *η*^2^ = 0.140) for which higher values were found for the social compared to no-social frame (Figure 2A). No other significant effects were detected for the SCR index. 

No significant effects were detected for the SCL index.

#### 3.1.2. HR

For the HR index, a first significant main effect was observed for Condition (*F* [1, 25] = 6.009, *p* = 0.022, *η*^2^ = 0.194) for which higher values were shown for the focus compared to the no focus condition.

A significant interaction effect regarding Condition × Frame was detected (*F* [1, 25] = 14.930, *p* = 0.001, *η*^2^ = 0.374) (Figure 2B). Pairwise comparisons showed higher HR mean values in the focus condition compared to the no focus condition in the no social frame (*p* = 0.004) and significantly higher mean values in the social frame compared to the no social frame in the no focus condition (*p* = 0.041). A second significant interaction effect Task × Frame was detected (*F* [1, 25] = 9.944, *p* = 0.004, *η*^2^ = 0.285) (Figure 2C). Pairwise comparisons displayed significantly higher mean values in the motor task compared to the linguistic task in the social frame (*p* = 0.007). For the HR index, no additional significant effects were observed.

#### 3.1.3. HRV

For HRV index, a significant interaction effect Condition × Task was detected (*F* [1, 25] = 8.767, *p* = 0.007, *η*^2^ = 0.260) (Figure 2D). Pairwise comparisons showed significantly higher mean values in the focus condition compared to the no focus condition in the motor task (*p* = 0.004) and significantly higher mean values in the linguistic task compared to the motor task in the no focus condition (*p* = 0.008). No other significant effects were found for the HRV index.

### 3.2. Second Order of Results

#### 3.2.1. BVP

For the BVP index, a significant main effect was observed for Frame (*F* [1, 15] = 5.129, *p* = 0.039, *η*^2^ = 0.255) for which higher values were found for the no social compared to the social frame. There were no further significant effects discovered for the BVP index.

#### 3.2.2. PVA

**Figure 3:**
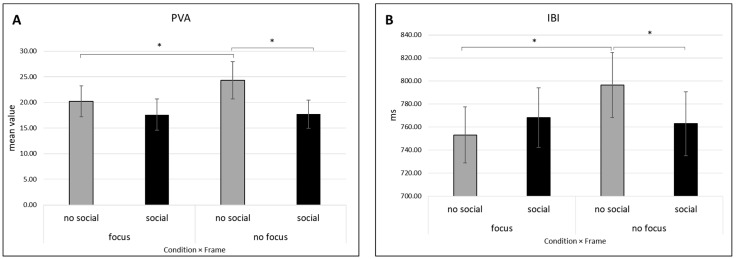
Second order of autonomic results: PVA and IBI. (**A**) Significantly higher PVA values can be observed in the no focus compared to the focus condition in the no social frame. Also, for the no focus condition, there were significantly higher mean PVA values in the no social compared to the social frame. (**B**) In the chart, significantly greater IBI values in the no focus condition compared to the focus condition in the no social frame, and in the no social frame compared to the social frame in the no focus condition, can be observed. Bars represent the standard error (SE) of ±1 for all plots; asterisks (*) denote statistically significant differences with *p* < 0.05.

For the PVA index a first significant main effect was found for Condition (*F* [1, 15] = 4.653, *p* = 0.048, *η*^2^ = 0.237) for which higher values were found for the no focus compared to focus condition. A second significant main effect was found for Frame (*F* [1, 15] = 5.091, *p* = 0.039, *η*^2^ = 0.253) for which higher values were found for the no social compared to social frame. Moreover, a significant interaction effect Condition × Frame was detected (*F* [1, 15] = 4.627, *p* = 0.048, *η*^2^ = 0.236) (Figure 3A). Pairwise comparisons showed significant greater mean PVA values in the no focus compared to the focus condition in the no social frame (*p* = 0.027). Also, for the no focus condition, there were significantly higher mean values in the no social compared to the social frame (*p* = 0.026). There were no additional significant effects detected for the PVA metric.

#### 3.2.3. IBI

Regarding the IBI index, a significant interaction effect regarding Condition × Frame was detected (*F* [1, 25] = 10.645, *p* = 0.003, *η*^2^ = 0.299) (Figure 3B). Pairwise comparisons displayed significantly greater mean values in the no focus condition compared with the focus condition in the no social frame (*p* = 0.007) and significantly greater mean values in the no social frame compared to the social frame in the no focus condition (*p* = 0.045). There were no additional significant effects detected for the IBI metric.

## 4. Discussion

By integrating ANS measurements in the field of social interoception, this study aimed at investigating the impact of the IA and social framing modulation on ANS reactivity during basic interpersonal synchronization tasks requiring motor and linguistic synchronization. Participants were required to perform two synchronizations tasks (linguistic versus motor synchronization tasks) under a focus and no focus on the breath condition (explicit versus implicit IA modulation). Moreover, the social context manipulation was operationalized by stressing the shared intentionality during the synchronization tasks (i.e., by applying or not a social frame to the synchronization task).

Study findings can be resumed in two orders of autonomic results based on the two main experiment conditions: the focus and no focus on the breath condition and the social and no social frame presence. The effects observed for SCR, HR and HRV can be considered as a first order of results mainly related to the focus condition and the social framing manipulation. First, higher SCR values were found for the social compared to no social frame. Greater HR values were found for the focus compared to the no focus condition as well as in the motor task compared to the linguistic task in the social frame condition. Finally, significantly higher HRV mean values were found in the motor task when there was the focus as opposed to when there was no intentional focus on the breath.

Meanwhile, the second order of autonomic results was related to the no focus and no social frame conditions: higher PVA and IBI values were found in the no focus compared to the focus condition where the tasks where not socially framed. Also, greater BVP values were found for the no social compared to the social frame scenario.

Starting from the first order of results, previous works on cooperation during social interactions found that in conditions with strong emotional involvement, where cooperative motivation was generated by providing feedback that reaffirmed the beneficial effects of the interpersonal interaction, SCL and SCR indices as well as HR rose [26]. In line with this evidence, in the present study, SCR values increased during social framing, that is when shared intentionality with the partner was emphasized, likely indicating increased engagement in the task. Similarly, HR showed an increase when especially the motor confronted with the linguistic synchronization task was socially framed, and that may as well indicate a condition of engagement toward the task. In a previous study, exploring the ANS in relation to gesture execution and observation, it was observed that the higher cognitive load required to carry out the motion seen in the video is what causes the encoder’s HR increase during the gesture simulation [35].

It should be underlined that limited former research considered autonomic modulation during joint synchronization tasks, and therefore, it is necessary to seek an interpretation of these indicators based on previous studies derived from similar contexts.

Taken together, this evidence highlights that the increase in SCR and HR could be considered as a reliable marker of arousal and engagement (regardless of the different or event opposite emotional dynamics), thus suggesting that in the present study, participants were more engaged when performing the synchronization tasks with an explicit and well-defined social frame: that is, the conditions in which social goal and empathic responsiveness were most expected. Moreover, in line with our hypothesis [8], this effect was significantly evident in the ANS system for the motor synchronization task. Research has shown that toddlers had a significant incentive to synchronize while drumming with social partners. They were also able to change their drumming pace and accuracy more accurately when drumming with a social partner as opposed to using a metronome [36]. According to the authors, drumming with a social partner induces a particular human urge to synchronize during cooperative rhythmic action [36].

In addition, present findings showed a task-independent increased of the HR in the focus compared to no focus condition, and there was a specific increased HRV in the focus confronted with the no focus condition while performing the motor synchronization task. Greater HRV has been linked to better stress and arousal regulation capacities [8]. Greater HRV is linked to a higher ability to control emotions [37,38], metacognitive awareness and mind reading [33], empathy [39], and with improved results on a number of memory, attention and inhibition tasks [40,41]. Therefore, it might be plausible that the condition in which participants were focusing on their breath and performing the motor synchronization tasks was the elective condition in which the IA manipulation was more effective in promoting physiological regulation during interpersonal synchronization.

Regarding the second order of results related to the no focus and no social frame conditions, one main result consists of higher PVA and IBI values in the no focus condition compared to the focus condition when the task was not socially framed. Also, higher BVP values were found for the no social compared to the social frame.

The observed increase in PVA in the no focus condition, particularly in the no social frame, might be due to the lack of emotional control on the situation. Indeed, in a previous study in the field of IA manipulation and empathy for pain, greater PVA values were found for painful confronted with non-painful hand stimuli regardless of IA manipulation. This result was interpreted as a physiological reaction of engagement toward the perceived effect of physical pain [24]. It is possible that performing a synchronization task without a request to pay attention to one’s body and without a specific social goal induced a situation where participants experienced a lack of control over the situation, which may have generated stress in the participants and a concomitant increase in PVA. In support of this possible interpretation, it can be noted that in terms of average trends, albeit in the absence of significance, the condition of social framing manipulation (in which the goal is clear) is the one in which lower levels of PVA are observed. In line with this result for PVA, an increase in BVP was also observed in the no social frame. Since the BVP signal increases in response to negative emotions (pain, hunger, and fear) and drops in response to peaceful relaxation, it is commonly understood as a defensive reaction metric, which marks vasoconstriction and offers information on the activity of the sympathetic system [42].

Finally, differently from what hypothesized, an increase in IBI values was observed in the no focus condition compared to the focus condition when the task was not socially framed. The “social engagement system” is thought to include IBI variations during nonthreatening social interactions, which is in line with polyvagal theory [43]. This system permits slight variations in IBI in response to the needs of the social interaction. An improved behavioral coordination and perception of cohesiveness among group members was predicted in a prior study by the emergence of physiological coordination in IBI amongst group members [44], thus suggesting the relevance of this autonomic marker even in social synchronization dynamics. However, in our study, the no social frame condition was not comparable with this previous work, since we considered the individual ability to synchronize. Therefore, this variability may be due to synchronizing in a more or less socially stressed context, where the subjects had to pay attention to the cooperative task. The independence between IBI and HRV may be considered as a clear dissociation between a generic social context and the required cooperation where the focus was on the joint action more than on the social effect of the action itself.

### Limitations and Future Directions

Although this is one of the first studies attempting to investigate the impact of focus on one’s breath on ANS metrics, it also presents some caveats. One limitation concerns the sample size, obtained through a convenience sampling method, and involving mainly university students, this limits the generalizability of our findings. Future studies should also consider integrating the autonomic measures employed in this work with other more specific indices, such as for example the calculation of the RSA that has been used in other studies [18,26]. Also, although this study focused on attention to breathing and not on voluntary control of breathing, the addition of respiration measures (performed with a respiration belt or videorecording) would promote the control of the voluntary component of the breath in the future.

Furthermore, the current research adopted a single subject approach, examining the effects of the breath attention and social framing manipulation on the individual participant’s ANS correlates. Nevertheless, by applying a dual subject approach, it could be interesting to investigate how IA affects the physiological connection between the dyad members as they synchronize with one another. With reference to the application of this experimental paradigm in hyperscanning studies, it is interesting to note that Tschacher and Meier (2020) [45] noted that clients and psychotherapists experience interpersonal physiological synchrony (especially breathing), and that this physiological linkage is positively linked with client blond and the therapist’s evaluation of the therapeutic session’s progress. Nevertheless, researchers did not explore if IA (as opposed to the volitional control on the breath) have an impact on physiological synchrony. Future studies should explore this link and might also consider the recruitment of specific dyads of participants (such as therapists and clients or by coaches and athletes) in which a social goal is clearly evident.

## 5. Conclusions

This work is, to the best of our knowledge, among the first ones to focus on the ANS reactivity during IA and social frame manipulation in the context of interpersonal synchronization. Our results suggested that individuals were more engaged (increase in SCR, HR and HRV) when performing the motor synchronization tasks with an explicit and well-defined social frame: that is, the conditions in which social goal and empathic responsiveness were most expected. On the other hand, when compared to the condition of interoceptive focus and social intentionality, performing a synchronization task without a request to pay attention to one’s body and without a specific social goal induced a situation of lack of control over the situation, which may have generated a sort of stress response (increase in higher PVA, BVP and IBI values) in the participants.

As practical implication, this study suggests that even brief breath awareness interventions (e.g., three minutes of IA manipulation) may promote a better psychophysiological response during motor synchronization tasks and can suggest potentially interesting spin-offs for intervention contexts that are based on mind and body practices (such as yoga or sports disciplines that require coordination with two people). In addition, thinking about the contexts in which motor synchronization sessions are foreseen (e.g., clinical motor rehabilitation, physiotherapy contexts), clarifying the social frame and goal at the beginning of the motor synchronization task would seem to favor a better psychophysiological bodily response.

## Data Availability

The datasets used and/or analyzed during the current study are available from the corresponding author upon reasonable request.
